# Salivary Extracellular Vesicles in Detection of Cancers Other than Head and Neck: A Systematic Review

**DOI:** 10.3390/cells14060411

**Published:** 2025-03-11

**Authors:** Wojciech Owecki, Karolina Wojtowicz, Kacper Nijakowski

**Affiliations:** 1 Student’s Scientific Group in Department of Conservative Dentistry and Endodontics, Poznan University of Medical Sciences, 60-812 Poznan, Poland; 86897@student.ump.edu.pl; 2 The Student Scientific Society, Poznan University of Medical Sciences, 60-806 Poznan, Poland; 3 Department of Histology and Embryology, Poznan University of Medical Sciences, 60-781 Poznan, Poland; kwojtowicz@ump.edu.pl; 4 Department of Conservative Dentistry and Endodontics, Poznan University of Medical Sciences, 60-812 Poznan, Poland

**Keywords:** saliva, extracellular vesicles, cancer, biomarkers, diagnosis

## Abstract

Cancer is one of the leading causes of death worldwide. Evidence indicates that extracellular vesicles are involved in cancer development and may be used as promising biomarkers in cancer detection. Concomitantly, saliva constitutes a non-invasive and inexpensive source of biomarkers. This systematic review investigates the use of salivary extracellular vesicles in detecting cancers located outside of the head and neck. PubMed, Web of Science, Scopus, and Embase were thoroughly searched from database inception to 16 July 2024. Data from sixteen eligible studies were analyzed, including glioblastoma, lung, esophageal, gastric, prostate, hepatocellular, breast, and pancreatobiliary tract cancers. The findings highlight strong diagnostic potential for lung and esophageal cancers, where specific exosomal RNAs and proteins demonstrated high accuracy in distinguishing cancer patients from healthy individuals. Additionally, biomarkers in glioblastoma showed prognostic value, while those in hepatocellular and pancreatobiliary cancers exhibited potential for early detection. However, gastric and prostate cancer biomarkers showed limited reliability, and breast cancer biomarkers require further validation. In conclusion, salivary extracellular vesicles present potential in non-invasive detection across multiple cancer types; however, their diagnostic power needs further research, including standardization and large-scale validation.

## 1. Introduction

Cancer is considered one of the most significant causes of death globally [1]. Although there is still a lack of potent therapeutics for the comprehensive cure of cancer, various treatment options are available, including surgery, radiotherapy, chemotherapy, gene therapy, stem cell therapy, targeted therapy, photodynamic therapy, precision medicine, and other methods [2]. Unfortunately, in many cases, cancer is diagnosed in an advanced stage when the probability of cure is relatively low [3]. Screening may enable early detection of cancer, offering a better prognosis for disease management [4]. Currently, cancer biomarkers are gaining increasing attention for their role in cancer diagnosis, monitoring the disease progress, relapses, or drug response, contributing to better survival rates and greater therapy efficacy [5,6]. In recent years, extracellular vesicles (EVs) have emerged as promising biomarkers for early cancer diagnosis, monitoring disease progression, or as novel targets in cancer treatment [7,8]. EVs are small, lipid membrane- bound vesicles released by cells into the extracellular space, facilitating intercellular communication [9,10]. On the other hand, intercellular communication is a pivotal feature of cancer, and recent evidence points to EVs as crucial mediators of this process via packaging and transfer of bioactive components to influence the function and biology of cancer and the tumor microenvironment cells [11]. Interestingly, reports indicate that EVs may regulate cancer initiation, growth, metastasis, or invasion [12]. Indeed, EVs contribute to cancer progression and metastasis via modulating immune systems, promoting angiogenesis, reprogramming fibroblasts, or conditioning the premetastatic niche [13]. EVs present in various body fluids are considered an unparalleled source of molecular features regarding the health status of the cells [14]. According to a recently published study [15], EVs are divided into several subtypes: exosomes, microvesicles, apoptotic bodies, supermeres, oncosomes, exomeres, and migrasomes.

All of them are membranous structures [16], but they are characterized by different features, contents, and modes of synthesis [17]. Extending this definition, exosomes (50–120 nm) arise via budding from endosomal membranes and contribute to tumor progression and immune evasion. Microvesicles (0.1–100 µm) are released directly from the cell membrane and can affect cancer cell survival. They also play a key role in cancer development by facilitating the formation of new blood vessels in an expanding tumor. Next, apoptotic bodies (0.8–5 µm), formed as by-products of programmed cell death, participate in interactions with immune cells in the tumor microenvironment (TME) [17,18]. Migrasomes (0.5–3 µm), recently identified as a distinct type of extracellular vesicle, are involved in cell migration and cancer metastasis. They contain various proteins and lipids that assist in cell movement and invasion into surrounding tissues. Oncosomes (1–10 µm) are vesicles produced by cancer cells that contain a variety of biomolecules, including proteins, nucleic acids, lipids, and organelles. Cancer cell-derived oncosomes may facilitate intercellular communication and provide a mechanism for cancer cells to manipulate their microenvironment, promoting tumor growth, metastasis, and immune evasion [17]. Exomeres and supermeres are small (<50 nm) particles containing lipids, nucleic acids, and proteins [19]. Supermeres are morphologically distinct from exomeres and are highly enriched with cargo involved in multiple cancers, including glycolytic enzymes, TGFBI, and miR-1246 [20].

A liquid biopsy is a powerful tool for the early diagnosis of numerous diseases, especially cancer. It can be performed to analyze a patient’s body fluids, such as urine, saliva, or blood. Liquid biopsies usually analyze circulating tumor cells (CTCs), circulating tumor DNA (ctDNA), or miRNA. However, EVs are increasingly being analyzed in these procedures [19]. These particles are secreted and taken up by receptor cells in organs via surface proteins or other endocytotic mechanisms [21]. They then enter different bodily fluids through several mechanisms, primarily via the circulatory and lymphatic systems [22]. The detection of extracellular vesicles in liquid biopsies allows for the efficient identification of genetic material derived from cancer cells or other biomarkers [21].

One of the several body fluids available to collect potential biomarkers is saliva [23]. The vast majority of saliva is secreted by the salivary glands, including the submandibular glands, parotid glands, and sublingual glands [24]. Moreover, saliva comprises secretions from the upper respiratory tract, plasma exudates, cell debris, gingival crevicular fluid, and components of microorganisms. This complex body fluid consists of various molecules, such as DNA, mRNA, miRNA, inorganic ions, proteins, and metabolites, which might serve as potential biomarkers in detecting disorders [25]. Saliva exhibits several advantages as a source of diagnostic biomarkers. The collection of saliva is non-invasive, repeatable, straightforward, and does not require specific skills. Additionally, saliva is readily accessible and highly durable, while its collection is low-cost and safe [26,27,28]. Indeed, salivary biomarkers have emerged in multiple disorders, such as cancers, endocrine, cardiovascular, autoimmune, gastrointestinal, and neurodegenerative diseases [24,29,30,31,32]. Some of these disorders may not seem evident to salivary diagnostics. Since saliva has a direct anatomical connection with the oral, endocrine, and digestive systems, diseases of these types might seem to be the most promising targets for salivary diagnostics. On the other hand, evidence indicates that other disorders that seem to exhibit no correlations with saliva also have their biomarkers present in this body fluid [33].

**Figure 1:**
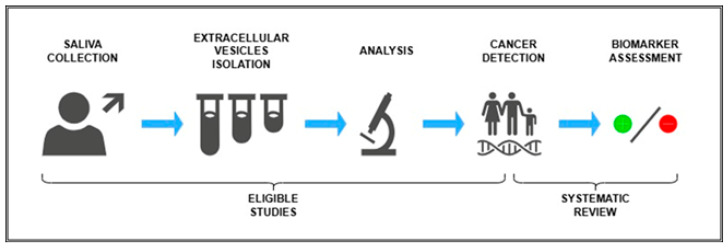
Study design of the systematic review. Eligible studies describe saliva collection, extracellular vesicles isolation, analysis, and potential in cancer detection. This systematic review investigates the potential use of salivary extracellular vesicles in cancer detection and assesses their utility as cancer biomarkers.

Considering the positive features of saliva collection and increasing interest in extracellular vesicles in cancer development, this systematic review was designed to highlight the use of salivary extracellular vesicles in the detection of cancers outside of the head and neck. The purpose of excluding head and neck cancers was to enable a more comprehensive analysis of cancers that might not seem to correlate with saliva. The general idea of this systematic review is presented in Figure 1.

## 2. Materials and Methods

### 2.1. Search Strategy and Data Extraction

Our systematic review was conducted based on the records published to 16 July 2024, according to the Preferred Reporting Items for Systematic Reviews and Meta-Analyses (PRISMA) statement guidelines [34], using the databases PubMed, Scopus, Web of Science, and Embase. The search queries included:

- for PubMed: saliva* AND ((extracellular vesicle) OR exosome OR microvesicle OR (apoptotic body OR apoptotic bodies) OR exomere OR migrasome OR oncosome OR supermere) AND (cancer OR carcinoma OR neoplasm OR tumour OR tumor OR oncolog*)
- for Scopus: TITLE-ABS-KEY (saliva* AND (“extracellular vesicle” OR exosome OR microvesicle OR (“apoptotic body” OR “apoptotic bodies”) OR exomere OR migrasome OR oncosome OR supermere) AND (cancer OR carcinoma OR neoplasm OR tumour OR tumor OR oncolog*))
- for Web of Science: TS = (saliva* AND (extracellular vesicle OR exosome OR microvesicle OR apoptotic body OR apoptotic bodies OR exomere OR migrasome OR oncosome OR supermere) AND (cancer OR carcinoma OR neoplasm OR tumour OR tumor OR oncolog*))
- for Embase: saliva* AND (‘extracellular vesicle’ OR exosome OR microvesicle OR ‘apoptotic body’ OR ‘apoptotic bodies’ OR exomere OR migrasome OR oncosome OR supermere) AND (cancer OR carcinoma OR neoplasm OR tumour OR tumor OR oncolog*).

Records were screened by the title, abstract, and full text by two independent investigators. Studies included in this review matched all the predefined criteria according to PECOS (“Population”, “Exposure”, “Comparison”, “Outcomes,” and “Study design”), as presented in Table 1. A detailed search flowchart is shown in the Section “Results”. The study protocol was registered in the international prospective register of systematic reviews, PROSPERO (CRD42024573878).

### 2.2. Quality Assessment of Included Studies

The risk of bias in each individual study was assessed according to the “Study Quality Assessment Tool” issued by the National Heart, Lung, and Blood Institute within the National Institute of Health [35]. These questionnaires were answered by two independent investigators, and any disagreements were resolved by discussion between them.

## 3. Results

### 3.1. General Information

**Figure 2:**
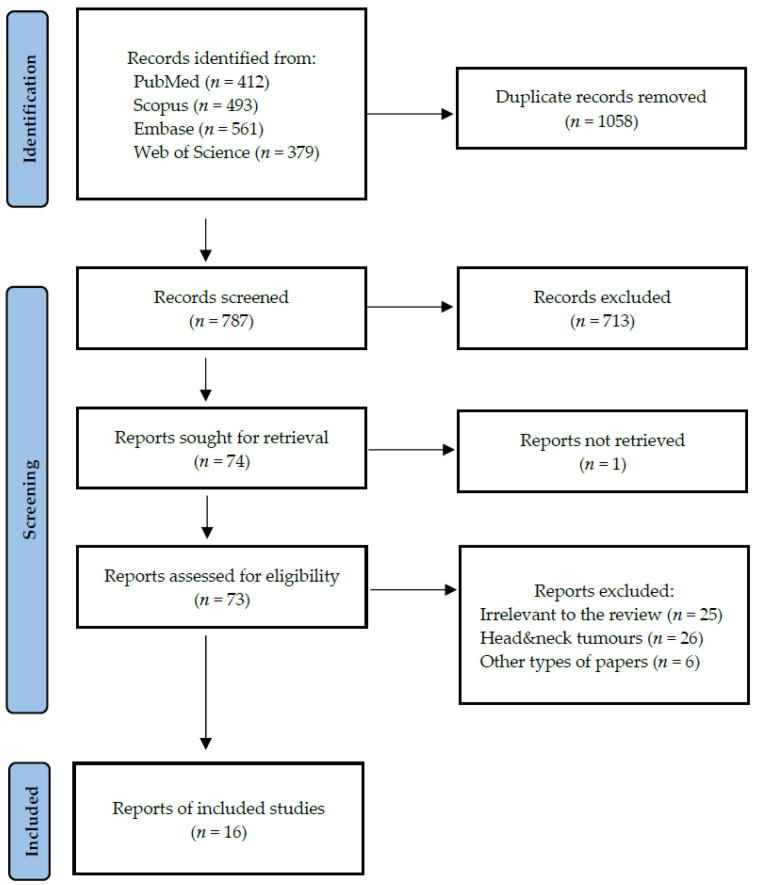
PRISMA flow diagram presenting search strategy.

Following the search criteria presented above, our systematic review included sixteen studies. All studies were published between 2016 and 2023. Figure 2 shows the detailed selection strategy of the searched records. In Table 2, we presented data regarding each eligible study included in this systematic review, which comprises year of publication, setting and involved participants, cancer diagnosis, inclusion and exclusion criteria, and TNM staging.

### 3.2. Participants, Cancer Diagnosis, and TNM Staging

In total, all eligible studies recruited 1452 cancer patients. One study did not report the number of enrolled cancer patients [47]. The vast majority of cancer patients were included in three studies involving esophageal squamous cell carcinoma (1290 participants) [37,38,39]. Seven studies investigated lung cancer, while gastric, prostate, pancreatobiliary, glioblastoma, hepatocellular, and breast cancer were investigated in single studies. Eligible studies demonstrate data collected in seven different countries. The majority of studies were performed in Asia (thirteen studies), especially in China (ten studies). TNM staging was provided in five studies; advanced cancer stages (III and IV) were more common than early stages (I and II) (728 and 510 patients; 50.14% and 35.12%, respectively, 214 not reported, 14.74%).

### 3.3. Inclusion and Exclusion Criteria

Some studies precisely defined inclusion and exclusion criteria. Nevertheless, these criteria differed between studies. Some patients were recruited with a new diagnosis without prior anti-cancer treatment. In several studies, controls were excluded if present with a history of malignancy, severe oral disease, diabetes, cardiovascular, renal, hepatic, and other disorders. Detailed inclusion and exclusion criteria are presented in Table 2.

### 3.4. Saliva Collection and Laboratory Methods

Details regarding saliva collection and laboratory methods were presented in Table 3. In most cases, saliva was collected in an unstimulated state, or the stimulation was not reported. One study investigated stimulated saliva [47]. If reported, approximately 5 mL of saliva were collected in most cases. Predominantly, sampling was performed in the morning hours (7 AM-12 noon). Usually, initial centrifugation was set at a range of 2000–3000× *g*, and the centrifugation time varied between 2 to 60 min, with the most common time being 15 min. Samples were stored at −80 °C in almost all cases. EVs isolation differed between included studies; some authors used commercial kits, whereas others adhered to protocols with various centrifugation and ultracentrifugation approaches. Similarly, EVs confirmation methods were heterogeneous; however, usually transmission electron microscopy (TEM), nanoparticle tracking analysis (NTA), and immunoblotting were used. The most commonly used method of biomarker identification was RT-qPCR (eight studies), followed by techniques based on mass spectrometry.

A recently published position paper provides guidelines regarding minimal information for studies of extracellular vesicles [56]. Recommendations for saliva-based studies suggest reporting the source of saliva, method of collection, and stimulus usage, as well as standardizing allowed drink and food intake prior to collection. Only a part of eligible studies include these information; in some cases, food and drink are not mentioned, or other factors are not clearly specified [36,51]. Furthermore, there are differences regarding the time from the last meal allowed [42,46] and the collection of stimulated or unstimulated saliva [43,47]. The aforementioned factors may affect saliva parameters, contributing to alterations in the results [56]. In addition, the isolation and confirmation of EVs differed between the included studies. A considerable heterogeneity regarding this aspect can be observed: some authors opted for differential (ultra)centrifugation protocols [36,44], others used acoustofluidic method [40], method based on affinity chromatography column combined with filter system [48], as well as commercially available kits utilizing membrane-based affinity binding [46], precipitation and targeted filtration for removal of protein contamination [49], differential solubility based precipitation [43], or polymer-precipitation [37,38,39,45,57]. As Welsh et al. [56] point out in their study, various methods have downsides that might affect EVs isolation quality. Differential (ultra)centrifugation is characterized by imperfect EV separations. Similarly, caution should be taken regarding commonly used commercial kits since not every kit may achieve EVs separation, or contaminants might be introduced [56]. Eligible studies also differ in the context of EVs confirmation method; however, in most cases, NTA, TEM, and immunoblotting were used.

### 3.5. Main Findings

Table 4 provides a study-by-study summary of findings on salivary EVs as cancer biomarkers beyond the head and neck.

### 3.6. Quality Assessment

**Figure 3:**
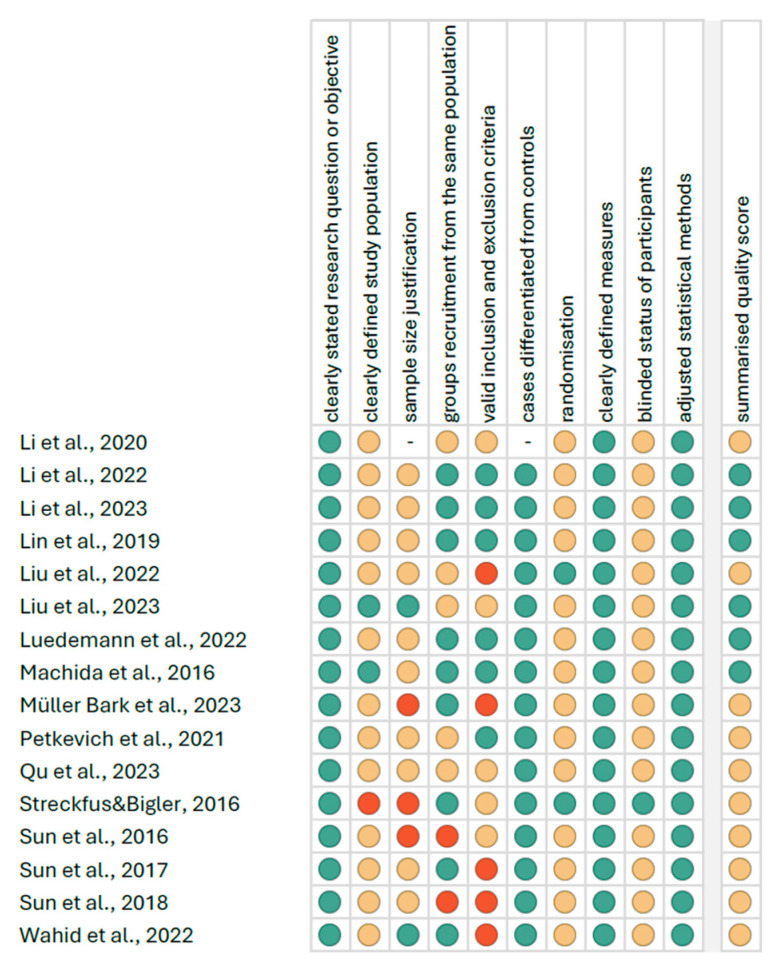
Quality assessment, including the main potential risk of bias (risk level: green—low, yellow—unspecified, and red—high and quality score: green—good, yellow—intermediate, and red—poor) [36,37,38,39,40,41,42,43,44,45,46,47,48,49,50,51].

Figure 3 reports the summarized quality assessment according to the “Study Quality Assessment Tool” issued by the National Heart, Lung, and Blood Institute within the National Institute of Health [35]. The most frequently encountered risks of bias were the absence of data regarding blinding (fifteen studies), sample size justification (thirteen studies, one study not applicable), randomization, and a clearly defined study population (fourteen studies). Critical appraisal was summarized by adding up the points for each criterion of potential risk (points: 1—low, 0.5—unspecified, 0—high). Six studies (37.50%) were classified as having “good” quality (≥80% total score), and ten (62.5%) were classified as “intermediate” (≥60% total score).

All of the included studies had the third or fourth level of evidence (case-control studies) according to the five-graded scale the classification of the Oxford Centre for Evidence-Based Medicine levels for diagnosis [58].

## 4. Discussion

### 4.1. Lung Cancer

Lung cancer is one of the most common cancers and the major reason for cancer-related deaths around the world [59,60]. Currently, over fifty histomorphological types of lung cancer are known; nevertheless, non-small cell lung carcinoma (NSCLC) and small cell lung carcinoma (SCLC) occur in the vast majority of cases [61]. On the other hand, NSCLC can be divided into multiple subtypes, including lung adenocarcinoma, lung squamous carcinoma, or large-cell lung cancer. Despite several treatment possibilities, the prognosis and survival rate remain poor [62]. Therefore, it seems that there is a crucial need for novel biomarkers to diagnose lung cancer at an early stage [63]. Over the years, numerous biomarkers have been proposed, including complement fragments, autoantibodies, circulating tumor DNA, DNA methylation, miRNAs, blood protein profiling, metabolomics, and others [64]. Growing evidence suggests that EVs impact the pathogenesis of lung cancer [65]. Cancer stem cell-derived EVs induce cancer progression via mediating angiogenesis, proliferation, metastasis, or drug resistance [66]. Evidence suggests that lung cancer EVs might promote tumorigenesis in normal cells and the proliferation of lung cancer cells [67]. Furthermore, EVs transfer a wide variety of molecules, including RNA, DNA, and proteins, which makes them a valuable source of potential lung cancer biomarkers [65,66,67].

In 2020, Li et al. [36] analyzed ultra-short circulating tumor DNA (usctDNA) in NSCLC patients utilizing both saliva and plasma. Since patients with NSCLC harbor the two front-line sensitizing epidermal growth factor receptor (EGFR) mutations (deletions in exon 19 and L858R in exon 21), the authors focused on this aspect. Three subtypes of EVs were investigated: apoptotic bodies, exosomes, and microvesicles. Exosomes comprised the majority of oncogenic EGFR mutations, which supports the hypothesis that exosomal usctDNA may be used as a potential tool for the prognosis or diagnosis of NSCLC.

Three years later, Liu et al. [41] investigated salivary biomarkers of early-stage lung adenocarcinoma patients. In this study, among the EVs, only exosomes were analyzed. Three miRNAs (miR-532-3p, miR-92b-5p, and miR-135b-5p) were additionally validated using RT-qPCR, confirming satisfactory results of the former method (RNA-sequencing). Almost five hundred putative target genes were found for the upregulated miRNAs. Further analysis indicated an association of these genes with cancer, suggesting that miRNAs encapsulated in salivary exosomes may play an important role in the diagnosis of lung cancer. Interestingly, the authors also investigated the performance of the three miRNAs mentioned above using an online database (The Cancer Genome Atlas—Lung Adenocarcinoma). AUC reached the highest values for all three miRNAs together and miR-135b-5p itself (0.912 and 0.906, respectively).

Wahid et al. [51] conducted a proteomic study of salivary EVs with a special focus on phosphoproteins in a group of six healthy and six NSCLC participants. Interestingly, some of these proteins (synaptosomal-associated protein 23, metalloproteinase inhibitor, G-protein coupled receptor family C group 5-member C, serpin B3, pyruvate kinase, and receptor-type tyrosine-protein phosphatase C) were associated with pivotal roles in cellular metabolism. Dysregulations in proline at position 2, threonine, and tyrosine at 16 were noted in patients with cancer. Concomitantly, phosphorylation on serine, tyrosine, and threonine was distributed significantly differently between the groups. Additionally, higher expression of threonine and tyrosine phosphorylation was observed in the experimental group. Upregulation of the RhoA signaling pathway, which plays an important role in oncogenic transformation, was noticed in the cancer group. Finally, the authors identified 11 and 19 distinctive salivary EV phosphoproteins and proteins (respectively) for NSCLC. Multiple observations included in this research may contribute to the development of salivary EVs biomarkers for NSCLC.

On the other hand, several studies investigated salivary EVs in patients with non-specified types of lung cancer. Qu et al. [46] recruited ten patients with lung cancer, as well as ten healthy individuals. Two lncRNAs were selected to determine their utility as biomarkers: *UC011CLY.2* and *NR_046326*, whereas *GAPDH* was used as the reference gene.

Another study [50] comprised six patients with lung cancer and six healthy individuals. In this case, proteomic analysis was applied. Proteins were investigated in salivary exosomes and microvesicles. Based on the participants’ status (healthy/lung cancer), 258 salivary EVs proteins were distinctive for lung cancer patients. Furthermore, several proteins were selected for validation: for microvesicles: CRNN, IQGAP, SPARCL1, and BPIFA1; whereas IQGAP, MUC5B, ENO1, and SPARCL1 were selected for exosomes. Among these EV proteins, BPIFA1, CRNN, MUC5B, and IQGAP showed significant differences between groups. Importantly, these differences disappeared when analyzed in the saliva itself, which suggests that salivary EVs can be used as a more specific source of biomarkers for lung cancer.

A similar approach was described in another study, with recruitment of a two times smaller sample. In contrast to the previous work, only exosomes were analyzed here. Among shared proteins, Mimecan, Cystatin-SA Transforming protein RhoA, Thrombospondin-1, Dipeptidyl peptidase 4, Protein lifeguard 3, Azurocidin, Tetraspanin, CD81 antigen, and Antileukoproteinase had a fold change greater than two in the experimental group. Comparison of data obtained from serum samples revealed 86 candidate exosomal biomarkers in both serum and saliva, while eleven of them repeated in both sources, supporting their role as lung cancer biomarkers (Ig lambda-7 chain C region; Vimentin; Phospholipid transfer protein, isoform CRA_c; Lactoperoxidase; Proteasome subunit alpha type; Annexin; Zinc-alpha-2-glycoprotein; Grancalcin; Cysteine-rich secretory protein 3; Protein S100; Myeloblastin; Trefoil factor 3; Calpain small subunit 1; and Histone H3) [49].

In an analogical way, another research analyzed salivary EVs in similar groups of participants to the above-mentioned study. Deepened research revealed that 80% of proteins were responsible for response to stimulus, whereas 60% were linked to multicellular organismal processes or stress response. On the other hand, 90% of these proteins had a molecular function related to protein, enzyme, or protease binding. Furthermore, 40% of these proteins were connected with the cancer network. Finally, 12 out of 63 proteins were associated with lung cancer, including Annexin family members (A1, A2, A3, A5, A6, A11), Mucin 1, Prominin-1, Nitrogen permease regulator 2-like protein, Histone H4, Carcinoembryonic antigen-related cell adhesion molecule 1, and Tumor necrosis factor alpha-induced protein 3. These conclusions support the potential use of these salivary EVs as promising biomarkers in lung cancer [48].

### 4.2. Esophageal Cancer

Esophageal cancer is characterized by poor prognosis and high mortality rate. It is considered the sixth leading cause of cancer-related death worldwide [68]. The most common type of esophageal cancer is the squamous cell type (ESCC), constituting 90% of all cases [69]. Multiple risk factors for developing esophageal cancer were identified, including poor diet, tobacco smoking, obesity, and alcohol consumption [70]. Since early diagnosis of this disease is necessary for effective therapeutic management, reliable biomarkers for early detection of this cancer are needed. In recent years, various potential biomarkers in different body fluids have been proposed, comprising proteins, cancer stem cells, or RNA-based biomarkers [71]. Accumulating evidence shows that EVs play a role in esophageal cancer pathogenesis and may serve as detection tools. EV-mediated molecules modulate intercellular communication in the tumor microenvironment and promote esophageal cancer progression [72,73]. On the other hand, alterations in various RNA subtypes provide a valuable profile of cancer pathology, implicating RNA use in cancer diagnosis [38,74].

In 2022, Li et al. [37] published a multicenter prospective study focusing on salivary exosomal RNAs as biomarkers for esophageal cancer. First of all, in a small group of participants, 1366 differentially expressed salivary exosomal small ncRNAs (sesncRNAs) (excluding miRNAs) were determined in patients affected by ESCC compared to controls, of which 32 were especially highly expressed, and the top five sesncRNA were selected for further investigation. Then, more participants were recruited, and the analysis revealed that only two sesncRNAs met a significance level: tsRNA (tRNA-GlyGCC-5) and a previously uncharacterized small non-coding RNA (called sRESE). On the other hand, the risk score of prognoses (RSP) was also investigated, and the bi-sesncRNA signature RSP was significantly correlated with histological differentiation and lymph node metastasis. Indeed, patients with high-RSP had significantly shorter overall survival and progression-free survival compared to patients with low-RSP. Notably, only patients with high-RSP adjuvant therapy had improved overall survival and progression-free survival. Both sesncRNAs seem to be promising biomarkers, not only for diagnosis, but also for prognosis and prediction of therapy in ESCC patients.

One year later, a similar study was published by Li et al. [38]. The performed ROC analysis revealed that all six miRNAs can be used as independent predictors; however, the best results reached a model combining all six miRNAs (AUC 0.968). In addition, all six miRNAs differed significantly in comparison to early-stage ESCC patients and healthy participants, and the combined model of all six miRNAs proved excellent results in identifying early-stage ESCC patients and controls (AUC 0.969). Based on all six miRNAs, a risk stratification model was established. High risk was significantly correlated with lymph node metastasis, greater tumor depth, histologic differentiation, higher probability of earlier death, overall survival, and progression-free survival. Indeed, this risk stratification model was an independent predictor of progression-free survival and overall survival. These findings suggest that the aforementioned salivary EVs miRNAs may serve as biomarkers for the early diagnosis and prognosis of ESCC.

On the other hand, another research focused on salivary exosomal GOLM1-NAA35 chimeric RNA (seG-NchiRNA) in ESCC patients [39]. Further analysis regarding surgical treatment revealed that seG-NchiRNA levels were significantly higher in patients with tumors that were larger in size. Moreover, a significant postoperative decrease in seG-NchiRNA levels was observed. Conversely, increased seG-NchiRNA expression after surgery was significantly associated with postsurgical recurrence. Investigation regarding chemoradiation showed that seG-NchiRNA levels were significantly lower after chemoradiation. Furthermore, alteration in seG-NchiRNA levels pre- and postchemoradiation significantly predicted clinical response and was an independent predictor of progression-free survival. This research implies that seG-NchiRNA may be considered a promising biomarker in ESCC diagnosis, prognosis, and response to treatment.

### 4.3. Gastric Cancer

Gastric cancer is the third most common factor leading to cancer-related death worldwide [75]. Since early-stage gastric cancer has a 95–99% 5-year survival rate compared to less than 30% in an advanced stage, early detection may contribute to the decrease of its mortality rate. Although endoscopic and radiological methods improve diagnostic possibilities, less invasive and time-consuming biomarkers, including recently investigated extracellular vesicles, circulating DNA, RNA, and proteins, may facilitate the screening process [76,77]. Importantly, EVs have also emerged as potential biomarkers in gastric cancer detection. Moreover, EVs play a crucial role in the development and progression of gastric tumors influencing intercellular communication [78].

In 2022, Liu et al. [40] investigated the potential noninvasive detection of gastric cancer using the composition information of collective Raman-active bonds inside small EVs illustrated by surface-enhanced Raman spectroscopy. An algorithm was developed based on machine learning to distinguish patients from cancers. Blood, saliva, and tissue-derived small EVs were analyzed. Relatively poor results of saliva-based small EVs in distinguishing patients with gastric cancer and healthy controls compared to blood and tissue suggest that the latter two sources are better choices.

### 4.4. Prostate Cancer

Prostate cancer is considered the most common noncutaneous cancer in males globally and the highest cause of cancer-related deaths among men in Western countries [79,80]. Early detection of prostate cancer currently focuses on harm minimization using multimodal techniques that combine MRI with serum prostate-specific antigen (PSA) [81]. Nevertheless, PSA remains the only screening biomarker and may be considered controversial due to its limitations; therefore, further study of potential biomarkers is of great importance [82]. Recent evidence shows that EVs may be considered potential prostate cancer biomarkers. Indeed, prostate cancer-derived EVs contribute to immune regulation, metastasis, and drug resistance in tumor pathology [83,84].

A study by Luedemann et al. [42] investigated 16 candidate exosomal miRNAs as possible biomarkers in the saliva of 43 prostate cancer patients and 32 non-cancer subjects. These results indicate that hsa-mir-200b and hsa-mir-331-3p exhibit potential as possible salivary EVs biomarkers for prostate cancer, although their accuracy is limited.

### 4.5. Pancreatobiliary Tract Cancer

The term ‘pancreatobiliary tract cancer’ encompasses malignant carcinomas in the gallbladder, pancreatic, and extrahepatic bile ducts [43]. Even though these carcinomas are relatively uncommon, their mortality rate remains high [85,86]. The detection of pancreatobiliary tract cancers is challenging because of their anatomical location and the lack of specific clinical symptoms [43,87]. Hence, developing novel and reliable biomarkers is necessary to provide early diagnosis and reduce fatality [43]. EVs might be considered a potential candidate since they encapsulate various messages and signal biomolecules characterizing cancer cells [88].

Four candidate salivary exosomal miRNAs were analyzed in a cohort of 12 patients with pancreatobiliary tract cancer and 13 healthy participants. Among cancer patients, two participants were affected by bile duct cancer, one person had gallbladder cancer, and the remaining nine were patients with pancreatic cancer. The performance of miR-1246 and miR-4644 in discriminating between pancreatobiliary tract patients and non-cancer participants reached satisfactory results, whereas the combination of both provided the best outcome (AUC 0.814, 0.763 0.833; sensitivity 66.7%, 75.0%, 83.3%; specificity 100%, 76.9%, 92.3%; respectively). Interestingly, significant correlations were observed between miR-1246 and cancer antigen 19-9 or between miR-1246 and miR-4644. This research indicates that both miRNAs may be considered as possible biomarkers for pancreatobiliary tract cancer [43].

### 4.6. Glioblastoma

Glioblastoma is considered the most common primary brain tumor in adults [89]. Moreover, glioblastoma is the most aggressive brain tumor, characterized by a very short time of patients’ survival [90]. Diagnosis of glioblastoma is usually based on radiological imaging and postoperative pathological findings. Nevertheless, these techniques have their limitations; therefore, the search for potential biomarkers may provide novel opportunities [91,92]. Considering that EVs impact cell-to-cell communication and the tumor microenvironment in glioblastoma, as well as carry a wide variety of proteomic or genetic cargo, EVs might facilitate glioblastoma detection [93].

A proteomic approach was utilized by Müller Bark et al. [44] to investigate salivary small EVs in a group of 18 glioblastoma patients and five healthy individuals. Samples were collected pre- and postoperatively, and patients were divided into two groups depending on their progression-free survival (favorable and unfavorable). Four proteins were selected for further investigation, namely 14-3-3 protein ε, aldolase A, enoyl CoA hydratase 1, and transmembrane protease serine 11B. All these proteins presented significantly decreased abundance in patients with poor outcomes. Aldolase A was also confirmed by Western blotting and further analyzed using the ROC curve in preoperative distinguishing between patients with favorable or unfavorable prognoses (AUC 0.955, sensitivity 90.91%, specificity 100.0%). This research provides insights into salivary small EVs as prognostic indicators for patients with glioblastoma and presents excellent results of aldolase A as a possible candidate.

### 4.7. Hepatocellular Carcinoma

Hepatocellular carcinoma is the third most common cause of cancer-associated death globally [94]. The main risk factors include HCV, HBV infection, diabetes, nonalcoholic fatty liver disease, and heavy alcohol consumption [95]. Although these factors are potentially preventable, the mortality rate of hepatocellular carcinoma is increasing [96,97]. Given the limitations of ultrasound techniques in recent screening recommendations, several novel promising biomarkers are emerging, which may overcome various barriers of current diagnostic methods [97]. Among them are EVs and miRNAs encapsulated in EVs, which have already been reported as potential HCC biomarkers in serum [98].

In 2021, Petkevich et al. [45] investigated salivary exosomal miRNAs in 24 patients with hepatocellular carcinoma, 24 patients with liver cirrhosis, and 21 healthy volunteers. The ROC analysis of a combination of all three miRNAs revealed satisfactory results in discriminating between patients with cancer and healthy controls (AUC 0.88, sensitivity 73%, specificity 100%). In contrast, the outcomes were poor in distinguishing between cancer patients and patients with cirrhosis (AUC 0.54, sensitivity 78%, specificity 50%). In conclusion, salivary exosomal miR-221-3p, 122-5p, and 21-5p might be considered potential biomarkers for hepatocellular carcinoma; however, their poor accuracy in differentiation between liver cirrhosis and hepatocellular carcinoma limits their use.

### 4.8. Breast Cancer

The most commonly diagnosed cancer worldwide is breast cancer [99]. Although breast cancer management has evolved recently, the mortality rate remains a significant problem [100]. Breast cancer is a dynamic and heterogeneous disease; therefore, identification of novel biomarkers may provide a source of valuable information regarding initial diagnosis or cancer progression monitoring [101]. EVs constitute a potential source of biomarkers with implications in breast cancer therapeutic effects and detection. The EVs proteome provides valuable information for breast cancer detection and stratification [102,103]. Ductal invasive carcinoma of the breast is the most common type of breast cancer, accounting for 70–80% of cases of all invasive types [104].

In 2016, Streckfus et al. [47] published a study concluding twenty-year research regarding altered salivary proteins in ductal invasive carcinoma of the breast. The authors divided altered proteins into various groups based on their cellular activity. Multiple proteins belonged to the molecular chaperones/heat shock proteins, cell growth-related (growth factor, epidermal growth factor receptors), immunity-related (immunoglobulins, peroxidases, S100), cytoskeleton-related (cytokeratins, carcin-embryonic antigen, gelsolin, moesin), metabolism-related (apolipoproteins, cystatins, kinases, alpha-enolase), and antimicrobial (cancer antigen 15-3, lactotransferrin, neutrophil defensin 3, histatine or mucin precursors) groups. Moreover, compounds such as annexins, calmodulin, ubiquitin, histone H4, and others were also deregulated. This study provides a valuable catalogue of altered proteins, which can be considered potential salivary biomarkers (including exosomal ones) for diagnosis of ductal invasive carcinoma.

### 4.9. Study Limitations

This study has some limitations. These include the heterogeneity of the included studies in terms of the diagnosis of various cancers, the types of salivary EVs investigated, and the inclusion or exclusion criteria for participants. Considering that some cancers were investigated in only one eligible study included in this systematic review, comparison and discussion were limited in these cases. On the other hand, cancers that repeated in eligible studies had diverse potential biomarkers, which hindered comparing their usefulness. The risk of bias was increased due to limited data regarding blinding, sample size justification, randomization, and a clearly defined study population. Furthermore, only some authors conducted and reported the results of ROC analysis to predict the reliability of potential salivary biomarkers in cancer detection.

## 5. Conclusions

Early detection of cancers constitutes a crucial need. According to our systematic review, salivary extracellular vesicles may be considered as potential candidates in cancer detection. Notably, some biomarkers provided excellent results in distinguishing between healthy controls and cancer patients. Moreover, several potential biomarkers were suggested not only for cancer detection but also for disease prognosis and therapy prediction. Nevertheless, further study involving more participants in different countries are needed to fully verify their usefulness in cancer diagnosis.

## Figures and Tables

**Table 1:** Inclusion and exclusion criteria according to the PECOS.

Parameter	Inclusion Criteria	Exclusion Criteria
Population	Patients aged 0–99 years, both genders	-
Exposure	Cancers other than head and neck	Head and neck cancers
Comparison	Healthy subjects	-
Outcomes	Salivary extracellular vesicles as markers	Other salivary alterations
Study design	Case-control, cohort, and cross-sectional studies	Literature reviews, case reports, expert opinion, letters to the editor, conference reports
	Indexed to 16 July 2024	Not published in English

**Table 2:** The demographic and clinical characteristics of included studies.

Author, Year	Setting	Study Group; (F/M), Age	Control Group; (F/M), Age	Diagnosis	Inclusion Criteria	Exclusion Criteria	TNM Stages
Li et al., 2020 [36]	China	4; (NR), (NR)	No controls	NSCLC	Patients: NSCLC	NR	NR
Li et al., 2022 [37]	China	4 cohorts; in total 376 (total NR), total NR	4 cohorts; in total 216 (total NR), total NR	ESCC	Patients: newly diagnosed ESCC without prior anticancer treatment	All: insufficient salivary exosomal RNA, technical problem on saliva collection; Patients: incomplete medical record; Controls: history of malignancy, severe oral disease, diabetes mellitus, renal or hepatic dysfunction, lung disease, severe immune alterations, cardiovascular event in the past 6 months	I-II: 144; III-IV: 196; 36 NR
Li et al., 2023 [38]	China	5 cohorts; in total 582 (total NR), total NR	5 cohorts; in total 319 (total NR), total NR	ESCC	Patients: newly diagnosed ESCC without prior anticancer treatment	All: insufficient salivary exosomal RNA, technical problem on saliva collection; Patients: incomplete medical record; Controls: history of malignancy, severe oral disease, diabetes mellitus, renal or hepatic dysfunction, lung disease, severe immune alterations, cardiovascular event in the past 6 months	I-II: 220; III-IV: 326; 36 NR
Lin et al., 2019 [39]	China	3 cohorts; in total 332 (total NR), total NR	3 cohorts; in total 98 (total NR), total NR	ESCC	Patients: newly diagnosed ESCC without prior anticancer treatment	All: insufficient salivary exosomal RNA, technical problem on saliva collection; Controls: history of malignancy, severe oral disease, diabetes mellitus, renal or hepatic dysfunction, lung disease, severe immune alterations, cardiovascular event in the past 6 months	I: 60; II: 69; III: 166; IV: 27; 10 NR
Liu et al., 2022 [40]	South Korea	15; (NR), NR	15; (NR), NR	gastric cancer	Patients: gastric cancer	NR	NR
Liu et al., 2023 [41]	China	18 (8/10); 50–75	18 (8/10); 50–75	lung adenocarcinoma	Patients: lung adenocarcinoma	oral diseases	I: 7; II: 9; III: 2
Luedemann et al., 2022 [42]	Germany	43; (43/0), 69.32 ± 8.82	31; (31/0), 66.96 ± 9.33	prostate cancer	All: men with elevated PSA-blood serum levels; Controls: men with elevated PSA levels without histologic proof of cancer; Patients: men with histologically verified prostate cancer	All: secondary diagnosis, infectious diseases, Sicca Syndrome, prior interventions such as operations of the salivary gland or prostate, urinary diversion with intestine tissue, radiotherapy of the pelvic region or antiandrogen therapy	NR
Machida et al., 2016 [43]	Japan	12; (6/6), 65 (45–84)	13; (7/6), 66 (53–83)	pancreatobiliary tract cancer (pancreatic, gallbladder, bile duct cancer)	Patients: pancreatobiliary tract cancer, before the onset of cancer therapy; Controls: >50 years of age and no history of cancer	Controls: diabetes, pulmonary diseases, cardiovascular diseases, kidney diseases, liver diseases, autoimmune diseases at the time of saliva collection	I: 0; II: 1; III: 1; IV: 10
Müller Bark et al., 2023 [44]	Australia	18; (9/9), 60 (37–82)	5; (3/2), 63.5 (58–71)	glioblastoma	Patients: glioblastoma	NR	NR
Petkevich et al., 2021 [45]	Russia	Patients with liver cancer: 24; (10/14), 49.5 (43–65); Patients with liver cirrhosis: 24; (9/15), 45.5 (35–55)	21; (9/12), 46.0 (30–60)	liver cancer	Patients with liver cirrhosis: liver cirrhosis, HCV status positive in anamnesis; Patients with liver cancer: liver cancer diagnosed by contrast-enhanced MRI with liver-specific contrast agents	Controls: hospitalization during last 12 months, cancer/autoimmune disease/drug addiction/alcoholism in anamnesis, pregnancy, lactation	NR
Qu et al., 2023 [46]	China	10; (NR), NR	10; (NR), NR	lung cancer	Patients: lung cancer with no primary surgical resection; Controls: healthy people who received routine health check-ups and showed no disease	NR	NR
Streckfus et al., 2016 [47]	USA	NR	NR	ductal invasive carcinoma of the breast	Patients: ductal invasive carcinoma of the breast	NR	NR
Sun et al., 2016 [48]	China	3; (NR), NR	6; (NR), NR	lung cancer	Patients: lung cancer	Controls: history of malignancy, immunodeficiency, hepatitis, autoimmune disorders, HIV infection	NR
Sun et al., 2017 [49]	China	3; (NR), NR	3; (NR), NR	lung cancer	Patients: lung cancer	NR	NR
Sun et al., 2018 [50]	China	6; (NR), NR	6; (NR), NR	lung cancer	Patients: lung cancer	NR	NR
Wahid et al., 2022 [51]	China	6; (3/3), 64.66 ± 3.44	6; (3/3), 66.16 ± 5.84	NSCLC	Patients: NSCLC	NR	NR

Abbreviations: ESCC, esophageal squamous cell carcinoma; HCV, hepatitis C virus; HIV, human immunodeficiency virus; MRI, magnetic resonance imaging; NR, not reported; NSCLC, non-small cell lung cancer; PSA, prostate-specific antigen.

**Table 3:** Saliva collection and processing.

Author, Year	Type of Saliva and Method of Collection	EVs Isolation and Confirmation	Initial Centrifugation and Storing	Method of Marker Determination	Potential Biomarkers
Li et al., 2020 [36]	NR	exosome isolation using differential centrifugation-based protocol as described elsewhere, record not retrieved [52]	centrifuged at 300× *g* for 10 min at 4 °C, followed by 2000× *g* for 20 min at 4 °C for apoptotic bodies, 16,500× *g* for 20 min at 4 °C for microvesicles, 120,000× *g* for 2 h at 4 °C for exosomes; EV pellets washed and resuspended in ice-cold PBS	EFIRM assay	exosomal EGFR exon 19-del and L858R mutations
Li et al., 2022 [37]	3–5 mL of unstimulated saliva collected by spitting into a 35-mm dish between 8 AM and 10 AM; no eating, drinking, using oral hygiene products for at least 1 h prior to collection	ExoQuick^TM^ exosomes precipitation solution used, mixed at 4 °C overnight, collected by centrifugation at 1500× *g* for 30 min, and 3000× *g* for 5 min, confirmed by TEM, immunoblotting, and NTA	centrifuged at 3000× *g* for 15 min at 4 °C, stored at −80 °C	RT-qPCR	tRNA-GlyGCC-5, sRESE
Li et al., 2023 [38]	3–5 mL of unstimulated saliva collected by spitting into a 35-mm dish between 8 AM and 10 AM; no eating, drinking, using oral hygiene products for at least 1 h prior to collection	ExoQuick^TM^ exosomes precipitation solution used, mixed at 4 °C overnight, collected by centrifugation at 1500× *g* for 30 min, and 3000× *g* for 5 min, confirmed by TEM, NTA, and immunoblotting	centrifuged at 3000× *g* for 15 min at 4 °C, stored at −80 °C	RT-qPCR, microarray	miR-1972, miR-4274, miR-4701-3p, miR-6126, miR-1268a, miR-4505
Lin et al., 2019 [39]	3–5 mL of unstimulated saliva collected by spitting into a 35-mm dish between 8 AM and 10 AM, 5 patients’ samples were collected at 3 time points of the day (9 AM, 3 PM, and 9 PM); no eating, drinking, using oral hygiene products for at least 1 h prior to collection	ExoQuick^TM^ exosomes precipitation solution used, exosomes precipitated by refrigeration at 4 °C overnight, collected by centrifugation at 1500× *g* for 30 min and for 5 min, confirmed by TEM, immunoblotting, and NTA	kept on ice during processing, centrifuged at 3000× *g* for 15 min at 4 °C, stored at −80 °C	RT-qPCR	seG-NchiRNA
Liu et al., 2022 [40]	5 mL of whole unstimulated saliva collected as described by Navazesh et al. [53]	acoustofluidic EVs isolation, confirmed by NTA and TEM	centrifuged at 2600× *g* for 15 min at 4 °C, stored at −80 °C	surface-enhanced Raman spectroscopy	composition information of the collective Raman-active bonds inside sEVs
Liu et al., 2023 [41]	approximately 5 mL of saliva collected, no swallowing or speaking	EVs isolation: centrifuged at 2500× *g* for 15 min at 4 °C, ultracentrifugation at 120,000× *g* for 2 h at 4 °C, re-centrifuged at 120,000× *g*, exosomes fixation and overloading onto a formvar/carbon-coated grid, confirmed by TEM, NTA, and immunoblotting	centrifuged at 2000× *g* for 5 min at 4 °C, stored at −80 °C	miRNA-seq, RT-qPCR	hsa-miR-4508, hsa-miR-4787-5p, hsa-miR-4488, hsa-miR-4492, hsa-miR-7704, hsa-miR-423-5p, hsa-miR-574-3p, hsa-miR-135b-5p, hsa-miR-1290, hsa-miR-34b-3p, hsa-miR-8485, hsa-miR-1246, hsa-miR-6516-3p, hsa-miR-92b-3p, hsa-miR-532-3p
Luedemann et al., 2022 [42]	5 mL of whole saliva collected, no smoking, drinking or eating for 2 h prior collection	EVs isolation: centrifuged at 1500 rpm for 10 min, then at 4700 rpm for 10 min, at 13,500 rpm for 15 min, at 160,000× *g* for 60 min at 4 °C, Trizol, chloroform added, rest for 3 min, centrifuged at 13,500 rpm for 15 min, incubated at −20 °C for 20 min, centrifuged at 13,500 rpm for 15 min, at 11,000 rpm for 5 min incubated at 60 °C for 15 min, followed by a brief centrifugation, confirmation NR	frozen at −20 °C, later stored at −80 °C, thawed at room temperature, and centrifuged at 1000× *g* for 2 min	delta-CT method, RT-qPCR	hsa-mir-200b, hsa-mir-331-3p
Machida et al., 2016 [43]	at least 0.5 mL of whole unstimulated saliva collected as described by Gallo et al. [54], collected in the morning (7 AM-12 noon) by spitting through a funnel into a tube kept on ice	exosomes isolation using Total Exosome Isolation Reagent (Invitrogen, Carlsbad, CA, USA), confirmation NR	stored at 4 °C for up to 6 h, then stored at −80 °C until use	RT-qPCR	miR-1246, miR-4644
Müller Bark et al., 2023 [44]	as described by Tang et al. [55]	isolation by differential centrifugation and ultracentrifugation (details unclear), confirmed by NTA, TEM, immunoblotting	as described by Tang et al. [55]	(DDA)-MS, SWATH-MS, Western blot	favorable/unfavorable: aldolase A, 14-3-3 protein ε (1433E), transmembrane protease serine 11B (TM11B), enoyl CoA hydratase 1 (ECH1)
Petkevich et al., 2021 [45]	collected in 50 mL tube after 2 h of fasting	exosomes isolation with miRCURY Exosome Serum/Plasma Kit (Qiagen, Germany), after centrifugated at 8000× *g* for 10 min and at 10,000× *g* for 30 min, filtrated, incubation at 4 °C for 1 h and centrifugation at 500× *g* for 5 min at room temperature, confirmation with indirect method of photon cross-correlation spectroscopy, ELISA	centrifuged at 3000× *g* for 10 min at room temperature, stored at −80 °C	RT-qPCR	miRNA-21-5p, miRNA-122-5p, miRNA-221-3p, all normalized to the corresponding miRNA-16-5p
Qu et al., 2023 [46]	5 mL of saliva collected by spitting into a sterile collection tube between 8 AM and 10 AM, after gargling for 5 min, no eating, drinking, smoking, and other stimulants for 1 h prior collection	EVs isolated using a procedure modified from the exoRNeasy protocol described in the exoRNeasy Midi Handbook, confirmed by TEM, NTA, and immunoblotting	centrifuged at 2600× *g* for 30 min at 4 °C, then supernatants at 10,000× *g* for 30 min at 4 °C, stored at −80 °C	dPCR chip, RT-qPCR	UC011CLY.2; NR_046326
Streckfus et al., 2016 [47]	stimulated whole saliva collected into pre-weighed disposable plastic cups	NR	placed on ice, centrifuged at 4 °C for 5 min, stored at −80 °C	LC-MS/MS mass spectrometry, 2D-gel analysis, ELISA, Western Blot	71 altered proteins
Sun et al., 2016 [48]	whole saliva collected, pooled, kept on ice during collection	EVs isolated with ACCF, centrifuged at 20,000× *g* for 1 h at 4 °C, filtered, centrifuged at 20,000× *g* for 1 h at 4 °C, confirmation with NTA and SDS-PAGE	centrifuged at 2600× *g* for 30 min at 4 °C, stored at −80 °C	LC-MS/MS	63 candidate proteins, especially: Annexin A1, A2, A3, A5, A6, A11; Nitrogen permease regulator 2-like protein (NPRL2); Carcinoembryonic antigen-related cell adhesion molecule 1 (CEACAM1); Prominin-1 (PROM1); Histone H4; Mucin 1; Tumor necrosis factor alpha-induced protein 3 (TNFAIP3)
Sun et al., 2017 [49]	saliva collected as described by Sun et al., 2016 [48], collected saliva-containing tubes were placed on ice	exosomes isolated by PureEXO^@^ isolation kit (101Bio, Mountain View, CA, USA), incubated for 10 min at room temperature, filtered, confirmed by TEM, NTA, and immunoblotting; prior isolation ACCF	centrifuged at 2600× *g* for 15 min at 4 °C, stored at −80 °C	LC-MS/MS based label free quantification	86 candidate proteins, especially: Ig lambda-7 chain C region; Vimentin; Phospholipid transfer protein, isoform CRA_c; Lactoperoxidase; Proteasome subunit alpha type; Annexin; Zinc-alpha-2-glycoprotein; Grancalcin; Cysteine-rich secretory protein 3; Protein S100; Myeloblastin; Trefoil factor 3; Calpain small subunit 1; Histone H3
Sun et al., 2018 [50]	unstimulated saliva collected, kept on ice during collection	EVs isolated through differential centrifugation: centrifuged at 10,000 or 20,000× *g* for 1 h at 4 °C, ultracentrifugated at 100,000 or 125,000× *g* for 2.5 h at 4 °C, confirmed by TEM, NTA, and immunoblotting; prior isolation ACCF	centrifuged at 2600× *g* for 30 min at 4 °C, stored at −80 °C	LC-MS/MS based label free quantification, Western blot	microvesicles: BPIFA1, CRNN; exosomes: MUC5B, IQGAP
Wahid et al., 2022 [51]	5 mL of saliva collected	EVs isolated by centrifugation at 7600× *g* for 40 min, ultracentrifugation at 110,000× *g* for 120 min, confirmed by by TEM, NTA, SDS-PAGE, and immunoblotting	centrifuged at 2600× *g* for 15 min, stored at −80 °C	LC–MS/MS-based label free quantification	30 distinctive phosphoproteins/proteins

Abbreviations: 19-del, deletion in exon 19; ACCF, affinity chromatography column combined with filter system; (DDA)-MS, data-dependent acquisition mass spectrometry; dPCR, digital polymerase chain reaction; EFIRM, electric field-induced release and measurement assay; EGFR, epidermal growth factor receptor; ELISA, enzyme-linked immunosorbent assay; EV, extracellular vesicles; Ig, immunoglobulin; LC-MS/MS, liquid chromatography-mass spectrometry/mass spectrometry; miRNA-seq, miRNA sequencing; NR, not reported; NTA, nanoparticle tracking analysis; PBS, phosphate-buffered saline; RT-qPCR, quantitative real-time polymerase chain reaction, quantitative reverse transcription polymerase chain reaction; SDS-PAGE, sodium dodecyl-sulfate polyacrylamide gel electrophoresis; seG-NchiRNA, salivary exosomal GOLM1-NAA35 chimeric RNA; sEVs, salivary extracellular vesicles; sRESE, small RNA identified in exosome from saliva of ESCC patients; SWATH-MS, sequential window acquisition of all theoretical mass spectra; TEM, transmission electron microscopy.

**Table 4:** Main findings from included studies.

Study	Diagnosis	Summary of Main Findings
Li et al., 2020 [36]	Non-Small Cell Lung Cancer (NSCLC)	The highest concentrations of usctDNA with EGFR exon 19-del and L858R mutation were observed in exosomes (*p* < 0.0001).
Liu et al., 2023 [41]	Lung Adenocarcinoma	The average size of exosomes was lower in the lung adenocarcinoma group compared to healthy controls (73.34 and 75.68 nm, respectively). Furthermore, the CD9 protein reached higher expression among patients with lung cancer, while the syntenin protein showed opposite results. Moreover, miRNAs were also investigated: seven miRNAs were downregulated and eight upregulated in the experimental group.
Wahid et al., 2022 [51]	Non-Small Cell Lung Cancer (NSCLC)	Seventy proteins were found to be upregulated, whereas 112 were downregulated. On the other hand, 333 and 524 sEV phosphopeptides were identified in NSCLC patients. Among 254 differentially expressed phosphosites in the cancer group, 37 were upregulated and 217 were downregulated. Moreover, di- and tri-phosphorylation sites’ expression was two times higher in NSCLC patients compared to healthy individuals.
Qu et al., 2023 [46]	Lung Cancer	The average levels of both lncRNAs were significantly higher in lung cancer samples compared to controls (*p* = 0.0001 and *p* = 0.0004, respectively).
Sun et al., 2018 [50]	Lung Cancer	In the experimental group 499 and 626 proteins were detected in salivary exosomes and microvesicles, respectively, whereas 650 and 642 were detected in controls, respectively. Based on the participants’ status (healthy/lung cancer), 258 salivary EVs proteins were distinctive for lung cancer patients. On the other hand, focusing on the types of EVs, 147 proteins were unique for lung cancer in exosomes and 284 in microvesicles. Concomitantly, 34 proteins were shared in both types of EVs. Label free quantification results revealed that in microvesicles, 134 proteins were downregulated and 109 proteins were upregulated, while in exosomes, 50 proteins were downregulated and 100 proteins were upregulated (fold change < 0.5, fold change > 2, fold change < 0.5, and fold change > 2, respectively).
Sun et al., 2017 [49]	Lung Cancer	A total of 222 and 238 proteins were identified in salivary exosomes of controls and lung cancer patients, respectively. Interestingly, 60% of these proteins were shared by both groups, whereas 97 proved to be unique for lung cancer.
Sun et al., 2016 [48]	Lung Cancer	Shotgun proteomic analysis enabled identification of 113 and 95 proteins in the lung cancer and control groups, respectively. Sixty-three proteins were distinctive for lung cancer patients.
Li et al., 2022 [37]	Esophageal Squamous Cell Carcinoma (ESCC)	sRESE and tRNA-GlyGCC-5 were significantly (*p* < 0.001) increased in the ESCC group compared with controls. The ROC analysis, performed in one of two cohorts (200 ESCC patients), proved satisfactory results (AUC 0.878 and 0.871, respectively, for tRNA-GlyGCC-5 and sRESE). Furthermore, the risk score of diagnosis was calculated for tRNA-GlyGCC-5 and sRESE, as well as the combined model of both (bi-sesncRNA); the latter combination reached the best results (AUC 0.933).
Li et al., 2023 [38]	Esophageal Squamous Cell Carcinoma (ESCC)	In the initial phases, which comprised 61 ESCC patients and 65 healthy participants, among 56 candidate salivary EVs miRNAs discriminating patients and controls, the six most promising were identified (2 upregulated: miR-4505 and miR-1268a, and 4 downregulated: miR-6126, miR-4701-3p, miR-1972, and miR-4274). Then, 521 ESCC patients and 254 healthy subjects were recruited for further investigation, which confirmed previous findings regarding six deregulated miRNAs.
Lin et al., 2019 [39]	Esophageal Squamous Cell Carcinoma (ESCC)	In the first phase (10 patients, 8 healthy controls), seG-NchiRNA levels were significantly higher in the experimental group compared to healthy participants. Interestingly, circadian variability was investigated, and no significant alterations were observed. In the second phase, additional participants were enrolled (322 patients), and the previously mentioned results were confirmed. Additionally, the ROC analysis proved satisfactory results in differentiating between ESCC patients and healthy subjects (AUC 0.912). Concomitantly, the ability to distinguish between early-stage ESCC patients and healthy controls was also observed, but with worse results (AUC 0.790).
Liu et al., 2022 [40]	Gastric Cancer	Salivary algorithm prediction accuracy reached 72%, which was less compared to blood and tissue (85% and 90%, respectively). Similarly, the “leave-a-pair-of-samples-out” analysis revealed that the performance of small EVs in saliva is much worse compared to tissue and blood (AUC 0.65 compared to 0.96 and 0.91, respectively).
Luedemann et al., 2022 [42]	Prostate Cancer	Two miRNAs, hsa-mir-200b and hsa-mir-331-3p, showed significantly lower concentrations in the experimental group compared to controls. The ROC curve analysis revealed reliable results of both miRNAs; however, the results were not excellent (AUC 0.663 and 0.648, sensitivity 81% and 74%, specificity 55% and 58%, respectively). The remaining 14 miRNAs levels did not differ significantly between the groups.
Machida et al., 2016 [43]	Pancreatobiliary Tract Cancer	The relative expression ratios (relative to U6 snRNA) of two miRNAs (miR-4644 and miR-1246) differed significantly between the groups—their ratios were significantly higher in the experimental group compared to healthy subjects.
Müller Bark et al., 2023 [44]	Glioblastoma	Score plots of the total salivary-EV proteome signatures showed clear differences between patients with unfavorable and favorable prognoses preoperatively. Furthermore, preoperatively, there was a significantly higher concentration of small EVs in patients with unfavorable outcomes compared to those with favorable outcomes. Before operation, two less abundant and 64 more abundant proteins were detected in patients with poor outcomes; postoperatively, ten less abundant and five more abundant proteins were identified in patients with unfavorable prognoses.
Petkevich et al., 2021 [45]	Hepatocellular Carcinoma	Among ten selected miRNAs for analysis, three (miR-221-3p, 122-5p, 21-5p) were determined in salivary exosomes. The exosomal-non-exosomal ratio of these miRNAs differed significantly between the cancer and control groups only in the case of miR-221-3p. The following investigation was performed with normalization to the miR-16-5p level. Two miRNAs (miR-221-3p, 21-5p) had significantly higher, while miR-122-5p had significantly lower, relative expression levels in cancer patients compared to controls and patients with cirrhosis.
Streckfus et al., 2016 [47]	Breast Cancer	Among 233 deregulated proteins in saliva, 71 were localized in salivary exosomes. On the other hand, 27 proteins were present in both salivary and breast tissue exosomes.

## Data Availability

No new data were created or analyzed in this study.
